# Editorial: Global excellence in translational immunology: Europe

**DOI:** 10.3389/fimmu.2023.1250624

**Published:** 2023-07-24

**Authors:** Federica Casiraghi, Norberto Perico, Giuseppe Remuzzi

**Affiliations:** Istituto di Ricerche Farmacologiche Mario Negri Istituto di Ricovero e Cura a Carattere Scientifico (IRCCS), Bergamo, Italy

**Keywords:** cancer, HLA, B cells, neoantigens, tumor-associated antigens, cancer metabolism

The goal of biomedical research is to understand biological systems so that we can determine the pathogenic mechanisms of disease and intervene in those processes. However, studying the human immune system is challenging. In this setting, scientists have relied heavily on mouse models and immortalized cell lines to tackle the fundamental questions on how the immune system works. These studies have yielded a deep understanding of how the components of the immune system function and have revealed genetic and biochemical mechanisms that underpin them. These achievements have also paved the way for the development of many immune therapies, from drugs/biologics to prevent allograft rejection to cytokine blocking biologics for the treatment of autoimmune diseases to immune checkpoint inhibitors for the treatment of cancers. Moreover, innovative technologies and tools now help to address the questions more salient to patients and their caregivers, for examples why there are differences in individual responses to a given immune therapy (1).

One of the major advances in the immunology field in the past decades has been the identification and characterization of immune checkpoint molecules, defined as ligand receptor pairs that exert inhibitory or stimulatory effects on immune responses (2). Most of these molecules are expressed on cells of the adaptive immune system, particularly T cells, and of the innate immune system. Checkpoint molecules with inhibitory activity, such as CTLA-4 and PD-1, are crucial for maintaining the self-tolerance and modulating the length and magnitude of immune responses of effectors in different tissues to minimize tissue/organ damage. That knowledge provided the rational for developing targeted immunotherapies, especially for patients with cancer.

The main premise of cancer immunotherapy is to (re)activate the immune system to specifically recognize and kill tumor cells. Among the most promising strategies, immune checkpoint inhibitors (ICI) and adoptive T-cell therapy have led to long-term survival benefits in patients with advanced malignancies (2). However, not all tumors appear to respond to these immunomodulatory therapies. This observation emphasizes the ability of tumors to escape the immune system through the loss of antigenicity and immunogenicity as well as through the establishment of an immunosuppressive microenvironment, characterized by accumulation of suppressive cellular and molecular components (3).

Against this background, this Research Topic provides a collection of contributions that describe recent pre-clinical and clinical progress in understanding the immune-escape mechanisms of tumors. Given the heterogeneity of the studies, in this Editorial we briefly describe and discuss some of the overarching themes of the seven articles that comprise the Research Topic.

Among the multiple mechanisms by which tumors escape immune surveillance, particularly relevant is the expression on their surface of ligands for the inhibitory immune checkpoint receptors. Engagement of these inhibitory receptors on activated T cells limits their survival and function and induces a sort of functional exhaustion, making them cells unable to exert anti-tumor immune response (4). The use of ICI has had a broad impact as a strategy to unleash tumor-specific cytotoxic T cells, with several antibodies targeting the axis Programmed Cell Death 1 (PD-1) -PD1 ligand (PD-L1) or the Cytotoxic T lymphocyte-associated antigen-4 (CTLA-4) approved as standard treatments for many solid tumors (2). However, ICI showed efficacy only in some patients or some indications. This is the case of triple-negative breast cancer (TNBC) where the use of anti-PD-1 and anti-PD-L1 antibodies reported conflicting results and the efficacy appears to be limited to a subset of patients (5). To identify possible strategies to complement ICI therapy, Boissière-Michot at al., collected 243 surgically resected TNBC specimens and evaluated the protein expression levels of TIGIT and its receptor PVR by immunohistochemistry. TIGIT (T cell immunoreceptor with immunoglobulin and ITIM domain) is an immune check point protein that is upregulated on activated T cells and NK cells. TIGIT binds to two ligands, poliovirus receptor (PVR, CD155) and nectin-2 (CD112) that are expressed by tumor cells and antigen-presenting cells in the tumor microenvironment (6). Both TIGIT and PVR were found to be highly expressed in most TNBC samples, mainly in stromal cells and malignant epithelium, respectively. TIGIT and PVR expression correlated with high tumor-infiltrating lymphocytes, including CD3^+^ and CD8^+^ positive cells as well as with PD-1 and PD-L1 positive cells. These data suggest that the axis PVR-TIGIT is an additional inhibitory checkpoint that limits adaptive and innate immunity and may represent a promising new target for the treatment of TNBC.

Parallel approaches to cancer immunotherapy focus on the stimulation of strong immune response that targets the expression of tumor-specific antigens, including neoantigens and tumor-associated antigens (TAA) (7). Neoantigens are mutant proteins that are expressed by cancer cells as the results of their genomic instability during oncogenesis. TAA, unlike neoantigens, are not only expressed by tumor cells but can be expressed at low levels by healthy tissues. Dependent on their expression pattern, TAA can be classified in cancer-testis antigens, differentiation antigens and antigens that are overexpressed in tumors (8). Neoantigens and peptides derived from a TAA can be presented in HLA molecules (MHCII or MHCI) to the T-cell receptor (TCR) of T cells, offering the opportunity to induce specific and long-lasting immune reactivity against the tumor. In their elegant mini review, Pagliuca et al., described how the somatic alterations of HLA heterogeneity can facilitate mechanisms of immune evasion that promote tumor growth and immune resistance. The high heterogeneity of human HLA molecules ensures the capability of the immune system to present a broad range of antigen peptides to effector T cells, thus conferring the competence to sustain the burden of diverse pathogens that could be encountered. Genetic alteration of this diversity may impair adaptive T cell response and provides an immune escape environment in cancer, infections, and possibly autoimmune diseases. Thus, the Authors recommend integrating into the clinical practice the analysis of HLA genotypic configuration and the relative dysfunction, which could have important prognostic implication in human diseases, especially in the immunology and onco-hematology fields.

An additional target to overcome tumor escape immune surveillance relates to post-translational modification of proteins, which is a source of tumor neoantigens during cellular stress and autophagy. In their paper, Brentville at al., found overexpression of citrullinated ER chaperon protein glucose-regulated protein 78 (GRP78) in murine and human tumor cell lines *in-vitro*, and in *in-vivo* grown melanoma tumor. After screening some citrullinated GRP78 peptides for the binding to HLA class II, they identified one peptide able to stimulate strong Th1 response and to delay melanoma growth in HLA-transgenic mice. CD4+ T cells able to recognize citrullinated GRP78 peptides were found in healthy individuals, suggesting that these cells have not been deleted or tolerized and exist in the normal immune repertoire. Therefore, citrullinated GRP78 can be a candidate for vaccination strategy in melanoma therapy. On the same line, Corrales et al. identified Lymphocyte Antigen 6 Family member G6D (LY6G6D), a member of the MHC class III leukocyte antigens, as TAA selectively expressed in a significant percentage of colorectal cancer (CRC) samples. Specific anti-LY6G6D antibodies were generated by immunizing mice with recombinant LY6G6D protein and two clones of antibodies were subsequently selected, the 10C1 for immunohistochemistry study and the clone 2C11A8 that the investigator employed for the construction of a specific anti-LY6G6D/CD3 T cell engager (TcE). In *in-vitro* experiments, anti-LY6G6D/CD3 TcE induced potent tumor cell lysis and the release of proinflammatory cytokines from fresh CRC slices. *In-vivo*, administration of TcE to PBMC-humanized NSG mice bearing LY6G6D+ CRC xenograft promote tumor regression. Interestingly, in 2D and 3D culture of CRC cells, T cells specifically activated by TcE were able to mediate bystander killing of neighboring LY6G6D-negative cells through a combined effect of IFNγ, TNFα and Fas/FasL pathway. Therefore, the TcE approach could be harnessed to activate cytotoxic T cells toward the TAA LY6G6D+ in CRC. The bystander killing activity of TcE may maximize and improve its therapeutic efficacy.

Cancer cells build all around themselves an immunosuppressive environment to promotes exhaustion of antitumor immune cells and the expansion of suppressor cells (9). For example, a massive peritumoral stroma surrounds pancreatic ductal adenocarcinoma (PDA) and appears to be responsible for the resistance to treatment and impaired drug delivery to the tumor mass. Ahmed et al. attempted to decipher the immune feature of the stroma in PDA patients by characterizing immune cells and cytokine release in stromal compartment and tumor epithelium. The analysis showed that two stromal cytokines, IL-9 and IL-18, were significantly associated with patient outcomes. IL-9 associated with improved patient survival and correlates with a network of Th9, Th1 and Th17 cytokines. On the opposite, stromal IL-18 associated with poor patient outcome and with a higher levels of exhausted T cells in PDA tissue. Nonetheless, further studies are needed to explore the potential of PDA stromal cytokines as prognostic markers or as novel therapeutic targets for the treatment of this cancer. Within the environment, tumors can also modify the immune cell metabolism by sequestering nutrients and by producing toxic waste compounds (10). This is well highlighted in the original research article by Pedrosa et al., who developed an immune metabolic signature (IMMETCOLS) on a training set of metastatic colorectal cancers samples which was then validated on The Cancer Genome Atlas (TCGA) database on tumors from 11 different origins. The IMMETCOLS signature classified tumors into three clusters with distinct metabolic and immune-suppressive profile: cluster 1 as mesenchymal-glycolytic tumors; cluster 2 as epithelial-non glycolytic tumor and cluster 3 as epithelial-glycolytic tumor. For each of these clusters, the authors suggest tailored ICI approach as well as combination of metabolic modulators. They also propose IMMETCOLS signature as a tool for the identification of novel immunotherapeutic approaches targeting the energy metabolism of cancer or of immune cells.

Finally, the Research Topic includes an interesting review by Meerhaeghe et al., that summarized all the available evidence on the cross talk between B cells and CD8+ T cells. It is now increasingly recognized that the role of B cell goes beyond the production of antibodies and more recent data suggest that B cells also promote pathogenic CD8+ T cell response *via* cross-presentation of antigen *via* MHCI and production of cytokines such as IL-27 and IL-15. In their review, the Authors provide an overview of studies demonstrating B cell modulation of CD8+ T cell function and the possible implications of this mechanism in controlling organ-specific and systemic autoimmune responses, infection diseases as well as anti-tumoral immunity, as well as the role of B cells in the formation and maintenance of CD8+ T cell memory, a controversial topic in translational immunology.

To conclude, the collection of article contributed to this Research Topic provides some excellent examples of recent advances which have deepened our knowledge of the complex mechanisms that tumors put in place to subvert the immune system to escape detection and destruction. It also highlighted novel target cellular pathways of potential innovative pharmacologic agents to improve therapeutic efficacy of anti-tumor treatments. Validation of the proposed approaches in future translational studies will be of high clinical significance. The final hope is that further research will be able to identify a universal mechanism/pathway of immune surveillance escape shared by the wide heterogeneous class of tumors so far characterized in humans.

## Author contributions

FC, GR, and NP contributed to the concept and writing of the editorial. They approved the manuscript for publication.

## References

[1] Buckner JH. Translational immunology: Applying fundamental discoveries to human health and autoimmune diseases. Eur J Immunol (2023), 2250197. doi: 10.1002/eji.202250197 PMC10600327 37101346

[2] Topalian SL Drake CG Pardoll DM. Immune checkpoint blockade: a common denominator approach to cancer therapy. Cancer Cell (2015) 27:450–61. doi: 10.1016/j.ccell.2015.03.001 PMC4400238 25858804

[3] Bhatia A Kumar Y. Cellular and molecular mechanisms in cancer immune escape: a comprehensive review. Expert Rev Clin Immunol (2014) 10:41–62. doi: 10.1586/1744666X.2014.865519 24325346

[4] Kalbasi A Ribas A. Tumour-intrinsic resistance to immune checkpoint blockade. Nat Rev Immunol (2020) 20:25–39. doi: 10.1038/s41577-019-0218-4 31570880 PMC8499690

[5] Chen X Feng L Huang Y Wu Y Xie N. Mechanisms and strategies to overcome PD-1/PD-L1 blockade resistance in triple-negative breast cancer. Cancers (Basel) (2022) 15(1):104. doi: 10.3390/cancers15010104 36612100 PMC9817764

[6] Chiang EY Mellman I. TIGIT-CD226-PVR axis: advancing immune checkpoint blockade for cancer immunotherapy. J Immunother Cancer (2022) 10(4):e004711. doi: 10.1136/jitc-2022-004711 35379739 PMC8981293

[7] Yarchoan M Johnson BA Lutz ER Laheru DA Jaffee EM. Targeting neoantigens to augment antitumour immunity. Nat Rev Cancer (2017) 17:209–22. doi: 10.1038/nrc.2016.154 PMC5575801 28233802

[8] Ward JP Gubin MM Schreiber RD. The role of neoantigens in naturally occurring and therapeutically induced immune responses to cancer. Adv Immunol (2016) 130:25–74. doi: 10.1016/bs.ai.2016.01.001 26922999 PMC6087548

[9] Jin M-Z Jin W-L. The updated landscape of tumor microenvironment and drug repurposing. Signal Transduction Targeted Ther (2020) 5:1–16. doi: 10.1038/s41392-020-00280-x PMC7447642 32843638

[10] DeBerardinis RJ. Tumor microenvironment, metabolism, and immunotherapy. New Engl J Med (2020) 382:869–71. doi: 10.1056/NEJMcibr1914890 32101671

